# Therapeutic Potential of Bioactive Compounds in Honey for Treating Osteoarthritis

**DOI:** 10.3389/fphar.2021.642836

**Published:** 2021-04-21

**Authors:** Carlos Martinez-Armenta, María Carmen Camacho-Rea, Gabriela Angélica Martínez-Nava, Rocio Espinosa-Velázquez, Carlos Pineda, Luis Enrique Gomez-Quiroz, Alberto López-Reyes

**Affiliations:** ^1^ Posgrado en Biología Experimental, Dirección de Ciencias Biológicas y de La Salud (DCBS), Universidad Autónoma Metropolitana Iztapalapa, Ciudad de México, Mexico; ^2^ Departamento de Nutrición Animal, Instituto Nacional de Ciencias Médicas y Nutrición Salvador Zubirán, Ciudad de México, Mexico; ^3^ Laboratorio de Líquido Sinovial, Instituto Nacional de Rehabilitación Luis Guillermo Ibarra Ibarra, Ciudad de México, Mexico; ^4^ Facultad de Ciencias de La Salud, Universidad Anáhuac México Sur, Ciudad de México, Mexico; ^5^ División de Enfermedades Musculo-esqueléticas y Reumáticas, Instituto Nacional de Rehabilitación Luis Guillermo Ibarra Ibarra, Ciudad de México, Mexico; ^6^ Área de Medicina Experimental y Traslacional, Departamento de Ciencias de la Salud, Universidad Autónoma Metropolitana-Iztapalapa, Mexico City, Mexico; ^7^ Laboratorio de Gerociencias, Instituto Nacional de Rehabilitación Luis Guillermo Ibarra Ibarra, Ciudad de México, Mexico

**Keywords:** Articular homeostasis, osteoarthritis, inflammation, honey flavonoids, Redox homeostasis, cartilage, chondroprotective activity

## Abstract

Dysregulation of joint tissue homeostasis induces articular degenerative changes and musculoskeletal diseases such as osteoarthritis. This pathology represents the first cause of motor disability in individuals over 60 years of age, impacting their quality of life and the costs of health systems. Nowadays, pharmacological treatments for cartilage disease have failed to achieve full tissue regeneration, resulting in a functional loss of the joint; therefore, joint arthroplasty is the gold standard procedure to cure this pathology in severe cases of Osteoarthritis. A different treatment is the use of anti-inflammatory drugs which mitigate pain and inflammation in some degree, but without significant inhibition of disease progression. In this sense, new therapeutic alternatives based on natural compounds have been proposed to delay osteoarthritis progression, particularly those agents that regulate articular homeostasis. Preclinical studies have shown a therapeutic application of honey and its bioactive compounds, ranging from treating wounds, coughs, skin infections, and are also used as a biological stimulant by exerting antioxidant and anti-inflammatory properties. In this article, we reviewed the current medicinal applications of honey with particular emphasis on its use regulating articular homeostasis by inhibiting inflammation and oxidative stress.

## Introduction

Osteoarthritis (OA) is a disabling condition characterized by joint degeneration; it is related to different etiological factors such as aging, genetics, metabolic and biomechanical stress. In this context, inflammation and oxidative stress play a pivotal role in the progressive deterioration of joint tissues including articular cartilage (AC), subchondral bone, synovial membrane and meniscus that maintain the functionality of joints until an exacerbated homeostatic dysfunction occurs (Minguzzi et al., 2018). Despite the clinical relevance of OA, which affects more than a quarter of the world population over 18 years of age (Chen D. et al., 2017), there are limited pharmacological strategies to prevent OA progression.

The management of OA includes weight control and specific physical exercises as interventional strategies to support the pharmacological therapy (Watt and Gulati, 2017). The first-line of intervention includes non-steroidal anti-inflammatory drugs and acetaminophen to control chronic pain. Cyclooxygenase-II inhibitors, intra-articular steroids and viscosupplementation are also considered when the standard treatment fails; nevertheless, their clinical efficacy is poor in patients with comorbidities (Jones et al., 2019). Therefore, the use of pain relief drugs neither represents a therapeutic strategy to halt or reverse cartilage damage, nor regulates the AC homeostasis, making AC prone to further damage (Saccomano, 2018).

Nowadays, beehive products are used to manage different inflammatory joint diseases as a non-pharmacological therapy. Under alternative or adjuvant therapeutic schemes, the potential physiopathological effect of honey, pollen, propolis and bee venom has been observed in humans (Almuhareb et al., 2019; Conrad et al., 2019), murine models (Owoyele et al., 2011; Hsieh et al., 2019) and *in vitro* (Jeong et al., 2015; Locatelli et al., 2018) studies. These health benefits are mainly observed when using honeys from the south hemisphere such as Manuka honey and stingless bee honey, and their health benefits are attributed to their pharmacological active constituents (Al-Hatamleh et al., 2020).

According to published data, different bioactive compounds commonly seen in honey have an effective role decreasing intra-articular injuries by inhibiting inflammation, oxidative stress, synovial hyperplasia and angiogenesis (Wang et al., 2007; Yang et al., 2018; Li et al., 2019; Orhan and Deniz, 2020; Yuan et al., 2020). Specifically, cartilage protection and enhancement of chondrocyte reparative functions induced by honey, involve several biologically active molecules such as chrysin, apigenin, quercetin, baicalin, luteolin, fisetin, butein, among other flavonoids and phenolic compounds. The present narrative review aims to discuss the emerging medical use of honey and to highlight the role of its polyphenols as potential regulators of articular homeostasis in OA. Therefore, we performed a search of published articles before March 2021 on PubMed database considering the following criteria: honey-derived flavonoids with biological effect on chondrocytes and articular homeostasis. From this search, we found that *in vitro* data is principally based on pure compounds, and only a few of them included assays in preclinical models of OA.

### Impact of Inflammation and Oxidative Stress in Cartilage Homeostasis

This degenerative joint disease is characterized by disruption of articular homeostasis, with a sustained production of pro-inflammatory cytokines, degradative enzymes of extracellular matrix (ECM), free radicals including reactive oxygen species (ROS) and reactive nitrogen species (RNS) (Surapaneni and Venkataramana, 2007).

**FIGURE 1:**
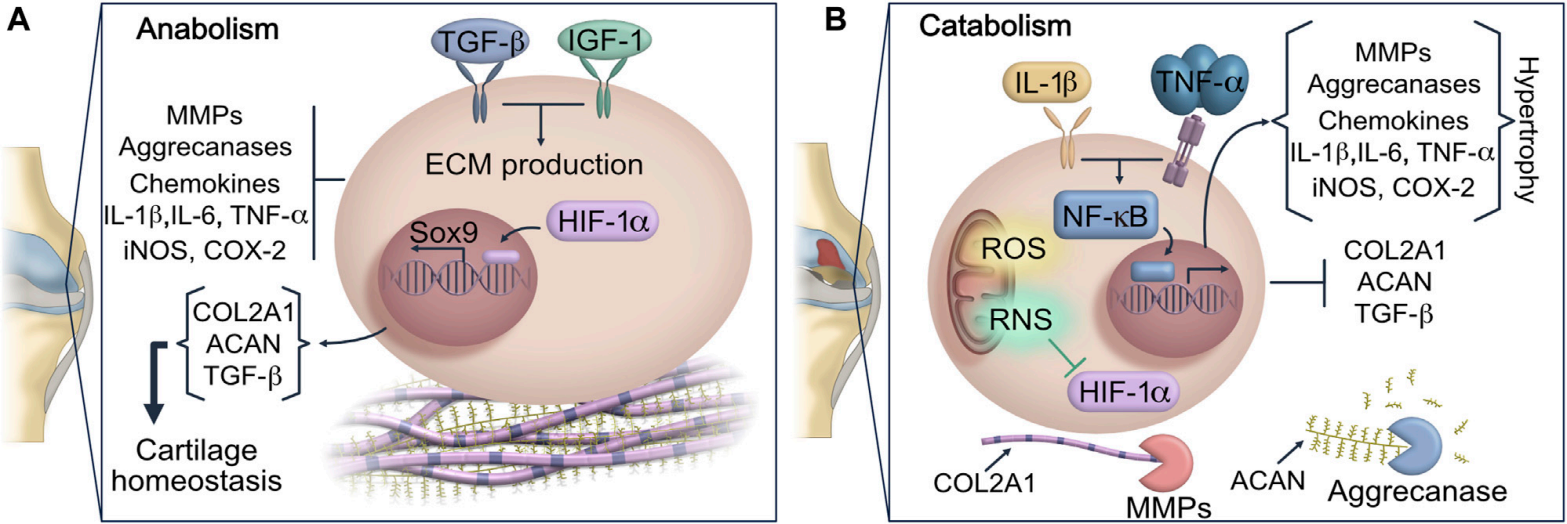
Dysregulation of joint homeostasis in OA. **(A)** Anabolic metabolism regulates gene expression and protein synthesis of COL2A1 and ACAN in chondrocytes, maintaining components of AC in a low-turnover state. **(B)** This scheme represents the homeostatic disruption of AC promoted by oxidative stress and pro-inflammatory cytokines. IL-1 and TNF-α signaling trigger the upregulation of MMPs and ADAMTS. Then the exacerbated release of MMP-13 prompts COL2A1 and ACAN hydrolysis, which are considered key components of AC matrix. The matrix degradation is intensified by the activity of ADAMTS, resulting in the loss of cartilage integrity and additional loss of joint function. Inflammation, ROS and RNS not only stimulate the expression of ECM degradative enzymes, but they also impair the chondrocyte ability to repair damaged cartilage by blocking HIF-1α and SOX9 signaling. AC: articular cartilage; ACAN: aggrecan; ADAMTS: a disintegrin and metalloproteinase with thrombospondin motif; COL2A1: type II collagen; ECM: extracellular matrix; HIF-1α: hypoxia-inducible factor 1 alpha; MMPs: metalloproteinases; ROS: reactive oxygen species; RNS: reactive nitrogen species.

Under normal conditions, chondrocytes exert anabolic functions that maintain a low-turnover replacement of specific ECM components including collagens, proteoglycans and non-collagen proteins (Singh et al., 2019). The rate of ECM protein deposition is regulated by the hypoxia-inducible factor 1 alpha (HIF-1α), which in a low oxygen concentration microenvironment induces the expression of SOX9, a master transcriptional regulator of chondrogenesis. SOX9 promotes the expression of chondrocyte-specific genes such as type II collagen (COL2A1) and Aggrecan (ACAN) which are the main ECM components in AC (Akiyama et al., 2002; Amarilio et al., 2007) (Figure 1). However, an altered functionality of the antioxidant system, unbalanced inflammation signaling, aging of AC and other adverse microenvironmental insults trigger a metabolic shift from anabolism to catabolism (Singh et al., 2019). The highly active metabolic state in AC leads to the synthesis of inflammatory and degradative proteins that activate cellular senescence and immune cell function inside the osteoarthritic joint.

The changes previously mentioned cause a state of sustained catabolism which perpetuate progressive destruction of cartilage and deteriorates joint tissues. Since the number of chondrocytes and their viability are substantially affected in OA, the synthesis of ECM components decreases. The impaired integrity of AC attenuates articular homeostasis, adversely affecting the function of other joint tissues such as synovium, meniscus, and subchondral bone (Stolberg-Stolberg et al., 2020). Moreover, due to the molecular stimuli triggered by collagen network damage and synovitis, the infiltration of mononuclear cells into synovium increases leading to sustained inflammatory signaling pathways. Furthermore, the enhanced inflammatory biomarkers in intraarticular space exert a prominent role in remodeling ECM including chondrocyte hypertrophic differentiation (Minguzzi et al., 2018).

The imbalance between anabolism and catabolism generated during OA progression can be slightly counteracted by the expression of the transforming growth factor-beta (TFG-β) which modifies the synthesis-replacement imbalance of proteoglycans in ECM (Scharstuhl et al., 2002; Jimi et al., 2019). However, the catabolic activity is also associated with a lower response to chondrocyte stimulation by insulin-like growth factor 1 (IGF-1), decreasing the production of ECM proteins and, consequently, the reparative process (Jimi et al., 2019; Morscheid et al., 2019).

#### Implications of Inflammation in Cartilage Degradation

Inflammatory mediators are regarded as critical players for cartilage destruction and synovitis in OA. However, genetic factors, aging-related changes and biomechanical stress due to obesity, surgery or a traumatic injury are the main cause of joint homeostatic dysregulation in OA (McAlindon et al., 2014). Additionally, the development, evolution and perpetuation of OA are characterized by a gradual loss of proteoglycans and COL2A1, followed by fibrocartilage formation which is linked to a high production of cytokines including IL-1β, TNF-α, IL-6, IL-15 and IL-18. The pro-inflammatory cytokine signaling stimulates a phenotypic shift in AC, apoptosis and aggravate synovial fibrosis (Jimi et al., 2019; Zhao et al., 2020). The exacerbated inflammatory stress in intraarticular space activates the canonical nuclear factor kappa-light-chain-enhancer of activated B cells (NF-κB) pathway in chondrocytes and synoviocytes. The NF-κB signaling is mediated by a multi-subunit IκB kinase (IKK) complex, which can respond to cytokine stimulus (Figure 1). Upon activation, NF-κB undergoes nuclear translocation, then it drives the expression of different genes including inducible cyclooxygenase 2 (COX-2), pro-inflammatory cytokines and chemokines that will uphold joint inflammation (Jimi et al., 2019). At molecular level, the high concentration of IL-1β and TNF-α in synovial fluid may activate catabolic processes driving fibroblast-like synoviocyte pyroptosis (Shen et al., 2014; Zhang L. et al., 2019). In the synovial membrane the exacerbated release of pro-inflammatory cytokines will induce an abnormal proliferation of synoviocytes triggering the infiltration of immune cells into synovial tissue. Additionally, the macrophage chemokine protein 1 (MCP-1) stimulates macrophages and neutrophils migration into the synovial space, which maintain**s** an exuberant inflammation level associated with the OA severity (Xu et al., 2015; Haraden et al., 2019).

High levels of IL-1β inside the joint induce the gene expression of Matrix Metalloproteinases (MMPs), of disintegrin and metalloproteinase (ADAM), as well as the gene expression of disintegrin and metalloproteinase with thrombospondin motif (ADAMTS) in chondrocytes and synoviocytes, accelerating the development of OA due to a mayor degradation of ACAN and collagen fibrils in cartilage (Struglics et al., 2006). Moreover, due to the high enzymatic activity in AC, the concentration of matrix degradation products including fragments of COL2A1, ACAN and fibronectin increases. This phenomenon triggers an upregulation of MMPs, VEGF and a high production of nitric oxide (NO) through the activation of TLR2 in chondrocytes that further promote catabolic function and cartilage destruction (Xie et al., 1993; Fichter et al., 2006; Hwang et al., 2015; Lees et al., 2015; Jung et al., 2019).

#### Oxidative and Nitrosative Stress in Cartilage Degradation

The repetitive cycles of inflammation and sustained anabolic-catabolic switch can cause an overproduction of ROS and RNS in cartilage, disrupting the intracellular redox status (Figure 1) that play an essential role in the regulation of chondrocyte hypertrophy, mitochondria dysfunction, as well as a role in oxidative damage to DNA, proteins and lipids (Ostalowska et al., 2006; Surapaneni and Venkataramana, 2007; Gavriilidis et al., 2013). In this context, oxidative stress modifies cartilaginous matrix proteins found in Golgi apparatus and endoplasmic reticulum of chondrocytes, decreasing their synthesis (Yu and Kim, 2013). Furthermore, the excess of ROS may also orchestrate ECM degradation *via* two different mechanisms. Firstly, ROS can exert direct hydrolysis of matrix components; secondly, it promotes the expression of MMPs that generates hypertrophic cartilage matrix (Lepetsos and Papavassiliou, 2016).

Previous studies have shown a decreased activity of antioxidant enzymes in OA, which impairs the metabolism and cell proliferation of chondrocytes (Morita et al., 2007; Surapaneni and Venkataramana, 2007; Goodwin et al., 2010). The redox balance in AC is affected by a down-regulation of Heme oxygenase 1 (HO-1) leading oxidative stress and consecutive senescence and apoptosis (Davidson et al., 2013; Cai et al., 2015; Takada et al., 2015). The nitric oxide (NO) concentrations above basal, function as a critical signaling molecule in hypertrophic differentiation and apoptosis of chondrocytes through a marked reduction of HIF-1α signaling (Bai et al., 2019). Furthermore, the homeostatic pathways related to chondrogenesis can be turnover due to the inhibition of HIF-1α (Figure 1).

Due to this complex pathophysiological scenery, apitherapy in OA has recently emerged as a novel non-pharmacological strategy to reduce the molecular events that drive structural and functional damage in joint tissues promoted by inflammation, nitrosative and oxidative stress in AC disorders.

### Anti-Inflammatory and Antioxidant Effects of Honey

Honey is a natural viscous sweet and flavorful solution consumed for its high nutritive value and its positive effects on human health. There are approximately 200 distinct chemical compounds in honey including a wide range of phenolic compounds that have antioxidant, bacteriostatic, antimicrobial and anti-inflammatory properties (Alvarez-Suarez et al., 2013). The biological effects produced by honey are attributed to its high concentration of polyphenolic compounds (flavonoids), which determine its antioxidant and anti-inflammatory properties (Shen et al., 2019; Goslinski et al., 2020). Honey contains different bioactive molecules including *p*-coumaric acid, eugenol, ferulic acid, caffeic acid, pinobanksin, pinocembrin, chrysin, quercetin, apigenin, galangin, isorhamnetin, gallic acid, kaempferol, syringic acid, luteolin and naringin; nevertheless, their concentrations vary depending on the type of honey (Ciulu et al., 2016; da Silva et al., 2016; Shen et al., 2019). In addition, a novel study has recently identified vesicle-like nanoparticles (VLNs) as a new bioactive agent in honey (Chen et al., 2021).

Honey has been used since ancient times as a therapeutic agent for a wide variety of clinical conditions. Its most remarkable effectiveness lies in wound healing (Frydman et al., 2020; Smaropoulos and Cremers, 2020) and treating gastrointestinal tract diseases (Bilsel et al., 2002; Miguel et al., 2017). Additionally, several studies have evaluated the effects of honey on cancer (Afrin et al., 2018b; Mohammed et al., 2020), diabetes (Sahlan et al., 2020) and dyslipidemias (Ramli et al., 2019), showing significant ameliorative effects. Likewise, antimicrobial effects have been reported when using honey (Rosli et al., 2020).

The role of honey in the inflammation process was reported by Al-Waili and Boni (2003); they demonstrated that by consuming diluted natural honey the concentration of prostaglandin E2, prostaglandin F2α and thromboxane B2 decreased in plasma of healthy individuals (Al-Waili and Boni, 2003). Another recent study has shown the biological role of honey-derived VLNs which exert a potent anti-inflammatory activity by suppressing NLRP3 inflammasome activation and NF-κB signaling. Furthermore, small-sized RNAs were identified as the molecules that remarkably ameliorated NLRP3 inflammasome activity, specifically, miR-4057 protected mice from acute inflammatory conditions in the liver (Chen et al., 2021). On other hand, different studies have explored the effects of honey intake on malondialdehyde and ROS levels in athletes and murine models, where individuals were subjected to high-impact exercise regimens. The authors concluded that honey consumption leads to a marked reduction of oxidative damage biomarkers generated by high physical activity (Ahmad et al., 2017; Jurcău and Jurcău, 2017; Hills et al., 2019).

Based on the results observed in preclinical studies, honey is a novel promise for the management of OA progression by targeting catabolism in joint tissues and recovering articular homeostasis.

### Potential Use of Honey for Articular Homeostasis Regulation

The protective activity of honey in OA has been evaluated in different animal models and *in vitro* studies. Prior research aimed to identify the anti-inflammatory effects of Manuka honey on macrophages stimulated with LPS. The results indicated that Manuka honey increases cell viability by decreasing apoptosis, inhibiting the production of free radicals and attenuating inflammation. These effects were regulated by decreasing caspase-3, p-p38 and *p*-Erk1/2 proteins at molecular level. Moreover, an increase of mitochondrial respiration and glycolytic activity were also demonstrated, which led to the expression and stimulation of *p*-AMPK, SIRT1 and PGC1alpha (Afrin et al., 2018a; Gasparrini et al., 2018). The biological potential of Manuka honey is attributed to the quercetin and gallic acid compounds, which are also present in other worldwide types of honey at high concentrations (Tomás-Barberán et al., 1993; Samarghandian et al., 2017).

On the other hand, an *in vivo* study noted the emerging anti-inflammatory and antioxidant capacity of Nigerian honey in formaldehyde-induced arthritis in Wistar rats. This study revealed that honey intake significantly reduced inflammation similarly to the treatment with indomethacin during a ten-day intervention (Owoyele et al., 2011). Furthermore, the beneficial effect exerted by honey and its derived bioactive compounds has also been described on bone health (Kamaruzzaman et al., 2019).

The flavonoids found in honey scavenge free oxygen radicals, reducing inflammation and minimizing tissue damage (Candiracci et al., 2012). A previous work by Alvarez-Suarez *et al.* analyzed the phenolic content of Manuka honey *via* HPLC-MS, and it was theorized that these components improve the intracellular antioxidant and anti-inflammatory response (Alvarez-Suarez et al., 2016). The efficacy of honey components has been recently explored in chondrocyte viability, inflammation and oxidative stress signaling.

### Honey Compounds Exert a Chondroprotective Effect

**FIGURE 2:**
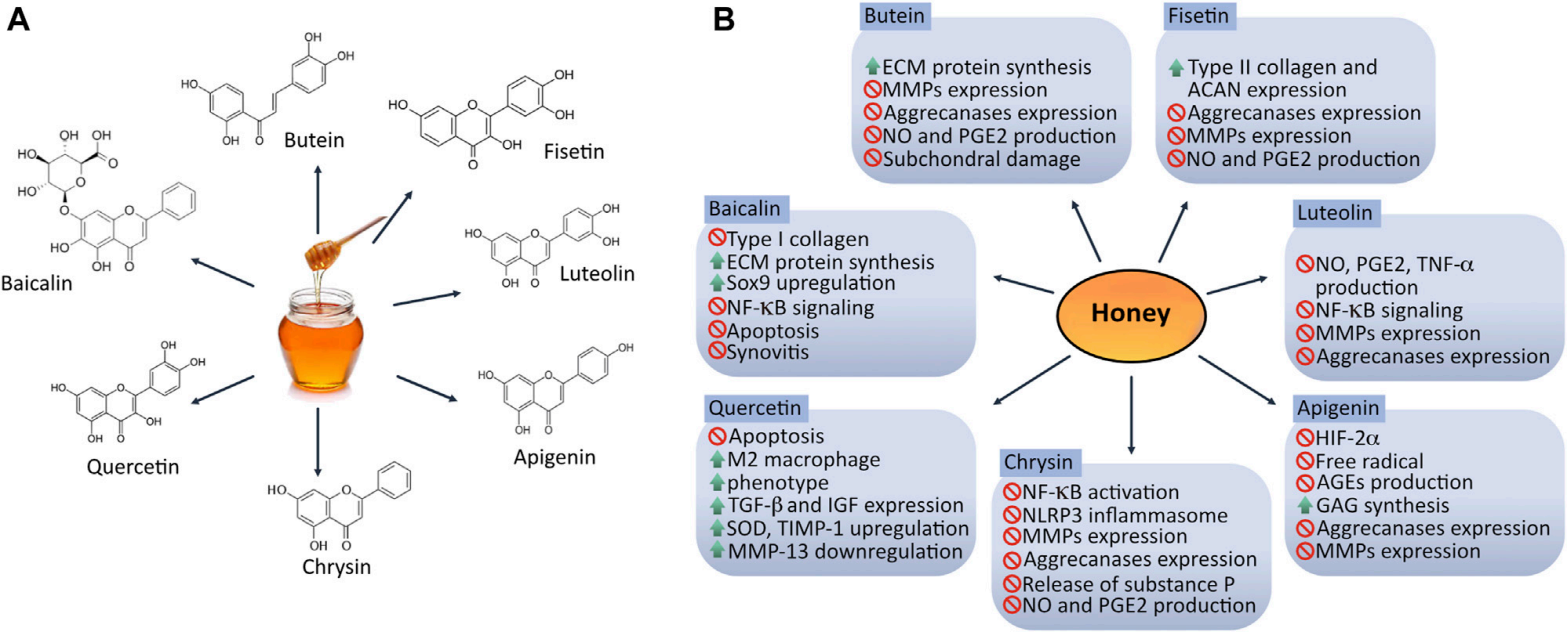
Chondroprotective effect of bioactive compounds found in honey. **(A)** Structure of honey bioactive molecules with potential application in targeting dysregulation of articular homeostasis. **(B)** Mechanisms exerted by honey-derived flavonoids in osteoarthritic joint. Different flavonoids found in honey can modulate catabolism in joint tissues *via* several signaling pathways promoting chondrogenesis-related genes expression such as SOX9, ACAN and COL2A1. ACAN: aggrecan; AGEs: advanced glycation end-products; ECM: extracellular matrix; GAG: glycosaminoglycans; HIF-2α: hypoxia-inducible factor 2 alpha; MMPs: metalloproteinases; NO: nitric oxide; PGE2: prostaglandin E2; SOD: superoxide dismutase. ↑ = up-regulate/stimulate/increase; ∅ = down-regulate/inhibit/suppress/reduce.

The chondrogenic potential of bioactive honey compounds has been observed in different *in vitro* and *in vivo* studies (Figure 2), suggesting promising applications of honey as an adjuvant therapy for repairing cartilage homeostasis specially by inhibiting inflammation and oxidative stress commonly seen in OA. Although information related to the mechanisms of action concerning articular homeostasis of each flavonoid found in honey is still limited, some of the biological processes underlying articular inflammation, oxidative stress, chondroprotection and metabolism in cartilage have already been investigated.

#### Effect of Honey Compounds on Articular Inflammation

Chrysin, a natural flavonoid extracted from honey was confirmed to attenuate NLRP3 inflammasome signaling, reducing synovitis and reducing the release of IL-1β, IL-18, substance *p*, and calcitonin gene-related peptide in monosodium iodoacetate (MIA)-induced knee OA model in rats (Liao et al., 2020). A second study has shown that chrysin dramatically blocked IL-1β-stimulated IκB-α degradation and NF-κB activation *in vitro* using IL-1β-injured human chondrocytes (Zheng et al., 2017b).

The biological activity of luteolin, another natural flavonoid, on signaling inflammation in chondrocytes has been recently documented. An *in vitro* study proved that a pretreatment with luteolin exerted an essential role targeting inflammation in rat chondrocytes *via* the inhibition of IL-1β induced NO, PGE2 and TNF-alpha production. In addition, luteolin reduced the phosphorylation of NF-kB that promotes the regulation of chondrocyte catabolic activity by decreasing the protein expression of iNOS, COX-2, MMP-1, MMP-3, and MMP-13 (Fei et al., 2019). Moreover, studies on animals have shown that gavage-administration (10 mg/kg/day for 45 days) in a MIA-induced model of OA has a protective effect attenuating AC destruction and OA progression (Fei et al., 2019). Another study evaluated luteolin biological activity modulating the catabolic activity in chondrocytes derived from a guinea pig model of OA; reporting that luteolin induced a downregulation of JNK, p38 and MMP-13, and a low production of inflammatory biomarkers including NO, TNF-α and IL-6 (Xue et al., 2019).

The efficacy of quercetin as an anti-inflammatory molecule was recently documented. Hu et al. demonstrated that quercetin suppresses inflammation by modulating synovial macrophages polarization to the M2 phenotype and inducing the expression of growth factors such as TGF-β and IGF, which promote chondrogenesis. The chondroprotective effect was also observed *in vivo*, using intraarticular administration of quercetin (Hu et al., 2019).

#### Effect of Honey Compounds on ECM Degrading Enzymes

The role of different flavonoids maintaining the synthesis of ECM components has also been described. The use of chrysin in an *in vitro* study showed favorable effects, suggesting that this flavonoid regulates the expression of MMP-1, MMP-3, MMP-13, ADAMTS-4 and ADAMTS-5, as well as the degradation of ACAN and COL2A1 on IL-1β-injured human chondrocytes (Zheng et al., 2017b). These findings are supported by a recent research where a protective effect was exerted by chrysin on human OA chrondrocytes *via* the suppression of the high-mobility group box chromosomal protein (HMGB1). It was demonstrated that chrysin increased the expression of COL2A1, while cell apoptosis, MMP-13 and IL-6 were inhibited (Zhang C. et al., 2019). Thus, chrysin may be a potential agent in the treatment of OA. Baicalin is another promising flavonoid found in honey with chondroprotective effects. Huang and colleagues recently reported that baicalin decreases IL-1β levels and suppresses the expression of collagen I, attenuating cartilage degeneration and promoting the proliferation of rabbit articular chondrocytes, as well as ECM restoration through COL2A1 and ACAN secretion *via* the upregulation of SOX9 gene (Huang et al., 2017).

The biological potential of fisetin and butein has also been studied. These compounds exert an anti-inflammatory and antioxidant effect by restoring the expression of COL2A1, ACAN and proteoglycans in monolayer cultures of chondrocytes. Fisetin and butein could also regulate the pro-inflammatory damage triggered by IL-1β through the induction of low NO and PGE2 production, as well as a significant inhibition of metalloproteinases and aggrecanases expression (Zheng et al., 2017a; Zheng et al., 2017c). Additionally, *in vivo* models of OA have shown less cartilage destruction and subchondral bone damage when mice are treated with butein and fisetin *via* intraperitoneal and oral gavage, respectively (Zheng et al., 2017a; Zheng et al., 2017c).

Luteolin has shown a potential role as a chondroprotective molecule. *In vivo* and *in vitro* studies have documented that luteolin inhibits gene expression and protein synthesis of MMP-1, MMP-3, MMP-13, ADAMTS-4 and ADAMTS-5 in cultured articular chondrocytes stimulated with IL-1β; furthermore, to analyze the direct effect of luteolin in rat joints, these animals received an intraarticular injection of luteolin, showing an inhibition of MMP-3 production prior stimulation with IL-1β (20 ng/30 μL) (Kang et al., 2014).

Apigenin is another honey compound that can play an essential role in AC homeostasis. A study demonstrated that apigenin decreases the expression of MMP-13 on IL-1β-treated human chondrocyte cell line SW1353 *via* signaling inhibition of c-FOS/AP-1 and JAK/STAT (Lim et al., 2011). Moreover, it has been reported that apigenin can inhibit the expression of HIF-2α, which is a master regulator of catabolic factors such as MMP-3, MMP-13, ADAMTS-4, IL-6 and COX (Cho et al., 2019). Recently, an anti-degenerative effect of apigenin was described by Park JS *et al.*, who reported that this molecule regulates the gene expression of matrix-degrading enzymes such as MMP1, MMP-3, MMP-13, ADAMTS-4 and ADAMTS-5 in rabbit chondrocytes. Additionally, it was observed that the MMP-3 production was inhibited in rats treated with apigenin plus IL-1β (Park et al., 2016).

#### Effect of Honey Compounds on Oxidative Stress and Antioxidant Mechanisms

In this context, a dysregulation of the nadase CD38 can impair articular chondrocyte homeostasis by promoting an excessive oxidative stress coupled with a significant decreased expression of Sirtuin-1 (SIRT-1). Nevertheless, some flavonoids as apigenin and quercetin can act as inhibitors of CD38 attenuating the release of NO and mitochondrial superoxide generation *via* maintaining function of SIRT-1 and SIRT-3, and regulating NAD^+^ decline on IL-1β-stimulated human chondrocytes (Kellenberger et al., 2011; Ansari et al., 2020).

Research using *in vitro* IL-1β-injured human chondrocytes also supports the biological role of chrysin inhibiting oxidative stress. Zheng et al. observed that chrysin significantly inhibits IL-1β-induced NO and PGE2 production on human chondrocytes that were pretreated and subsequently stimulated with the pro-inflammatory agent. Additionally, this flavonoid down-regulated the expression of COX-2 and iNOS (Zheng et al., 2017b).

Recently, a study reported that the role of quercetin is not limited to inhibit inflammation, for quercetin also promotes an anabolic activity on IL-1β-stimulated rat chondrocytes as well as an anti-apoptotic effect, *via* targeting ROS and inhibiting ER stress through the activation of SIRT1/AMPK signaling pathway (Feng et al., 2019; Hu et al., 2019). Furthermore, a second report showed that quercetin gavage-administered at 25 mg kg^-^1 in a rabbit model of knee OA up-regulates superoxide dismutase (SOD) and tissue inhibitor of metalloproteinases-1 (TIMP-1), promoting a downregulation of MMP-13 in synovial tissue (Wei et al., 2019). Thus, quercetin reduces tissue degeneration in OA by weakening oxidative stress responses and inhibiting the degradation of cartilage ECM.

There are different potential properties of apigenin. For instance, Crasci et al. reported that apigenin can be a free radical scavenger and a potent advanced glycation end-product inhibitor. They also showed that apigenin restored the glycosaminoglycans (GAGs) production when it was used for treating human articular chondrocytes previously stimulated with IL-1β (Crasci et al., 2018).

#### Effect of Honey Compounds on Chondroprotection

The biological activity of baicalin has been consistent in different studies, showing that baicalin protects chondrocytes from apoptosis and ECM degradation (Chen C. et al., 2017; Yang et al., 2018; Li et al., 2020). In this sense, Chen *et al.* identified baicalin as a potential candidate for OA treatment, as it prevented cartilage destruction and synovitis relief in OA *in vivo* models (Chen C. et al., 2017). Moreover, baicalin suppresses the expression of apoptosis-related genes induced by H_2_O_2_ (Pan et al., 2017; Cao et al., 2018), while induces COL2A1, ACAN and SOX9 expression in H_2_O_2_-treated chondrocytes (Cao et al., 2018).

It is well known that maintenance of autophagy is essential for preserving cartilage integrity. In this sense, a novel study documented that baicalin exerts an anti-apoptotic role through the up-regulation of Bcl-2 expression and through autophagy activation *via* miR-766–3p/apoptosis-inducing factor mitochondria-associated 1 (AIFM1) axis, which enhances ECM synthesis on human OA chondrocytes (Li et al., 2020). Similarly, Ansari et al. found that another flavonoid, butein, can activate autophagy in IL-1β-stimulated human chondrocytes by inhibiting the mechanistic target of rapamycin (mTOR) signaling (Ansari et al., 2018).

Considering the several health benefits and general well-being that have been associated with honey and its components, the emerging use of these products as a complementary strategy to regulate molecular mechanism underlying articular homeostasis is leading to further investigation in tissue engineering application for cartilage repair.

### Honey in Cartilage Tissue Engineering

The cartilage ability to self-regeneration is low, even when it is substituted with tissue-engineered constructs. However, many promising strategies are still attempting the promotion of AC repair and regeneration. In this regard, recent studies have focused on the development of honey-biomaterial based scaffolds such as hydrogels in order to prompt cartilage repair, due to the honey intrinsic antibacterial properties and its unique viscosity enhance the mechanical features of hydrogels (Abd El-Malek et al., 2017; Hixon et al., 2019; Bonifacio et al., 2020b). The innovative inclusion of Manuka honey into hydrogel promotes *in vitro* human mesenchymal stem cell chondrogenesis *via* increasing the expression of COL2A1 as well as the synthesis of GAGs and proteoglycans; additionally, no cytotoxic effect has been observed (Bonifacio et al., 2018; Bonifacio et al., 2020a). To date, *in vivo* studies of honey-contained hydrogels activity have shown significant results inhibiting infections and non-severe immunological reactions (Bonifacio et al., 2020a), which represent a promising tool for the regeneration of cartilage.

### Honey Biosafety for Clinical Applications

The increased interest in honey for medical use is leading to a strict regulation of its quality and safety. Honey can also contain toxic compounds including pesticides (Chiesa et al., 2018; El Agrebi et al., 2020), heavy metals (Bartha et al., 2020; Bosancic et al., 2020) and antibiotics (Barrasso et al., 2018) due to environmental pollution in honey harvesting areas. Bacterial contamination is another relevant factor that must be taken into consideration to ensure that honey is suitable for therapeutic purposes; for instance, the presence of *Clostridium botulinum* spores have been previously identified in honey samples (Nevas et al., 2002; Rosli et al., 2020). In this context, the use of medical-grade honey (MGH) guarantees its safety for clinical application (Hermanns et al., 2019). It has been proved that honey bioactivities as antimicrobial, wound healing, antioxidant and anti-inflammatory properties are still present after gamma radiation sterilization and the storage conditions are extended (Postmes et al., 1995; Molan and Allen, 1996; Hussein et al., 2014).

Although Manuka MGH is widely investigated as a novel non-pharmacological therapeutic strategy worldwide, other honey types are emerging with similar biological activity. In early reports, honey from stingless bees including *Melipona* spp., *Trigona* spp., *Tetragonisca* spp., and *Scapt*o*trigona* spp have exhibited therapeutic properties for treating inflammation (Ranneh et al., 2019; Biluca et al., 2020), wound healing (Abd Jalil et al., 2017; Abdul Malik et al., 2020) and oxidative stress (Abid et al., 2017; Ranneh et al., 2018; Biluca et al., 2020). Furthermore, the bioavailability of the most promising compounds such as apigenin, chrysin and quercetin has been previously reported in honey from stingless bees (Zulkhairi Amin et al., 2018). Therefore, they have the biological potential for modern medicinal applications in other pathologies related to dysregulated articular homeostasis.

## Conclusion and Future Perspectives

Since ancestral times, the therapeutic effects of honey have been described and widely observed in ameliorating the clinical course of wounds, coughs, skin infections and inflammation. Nowadays, the effectiveness of honey on counteracting articular damage to improve the quality of life of individuals with OA is being explored. We know that the bioactive compounds of honey exert chondroprotective activity by counteracting the homeostatic dysregulation of the joint. Therefore, its use as a therapeutic tool in the management of OA is widely supported, as it can shift major OA-related signaling pathways. This adjuvant non-pharmacological strategy might relieve pain, regulate joint homeostasis and repair AC, slowing down the OA progression; thus, reducing physical limitations, disabilities, mental stress and the socioeconomic burden commonly seen in individuals with this chronic disease.

There is a clear evidence that natural products represent an excellent source of bioactive molecules with potential medical applications. We introduced seven natural compounds derived from honey as possible candidates for treating OA due to their chondroprotective activity. However, there is a limited number of *in vitro* and *in vivo* reports showing the molecular pathways underlying the biological effect of honey-derived flavonoids. Articular homeostasis is quite complex, and its total restoration by a single molecule after either a biomechanical, inflammatory, or oxidative stress stimulus can be complex. Nevertheless, maximal therapeutic potential can be achieved by the combination of these molecules. However, suitable dosage and outcome represent a challenging issue. In this context, further preclinical studies are required to validate the honey emerging applications as a rational therapeutic strategy for OA, considering that most current reports have shown the effect of biologically active flavonoids on articular homeostasis regulation on *in vitro* research. Finally, it is highly relevant to develop clinical trials to verify the safety and efficacy of honey-derived bioactive compounds to better understand their activity at the cellular and molecular level for future therapeutic approaches.
